# ^1^H, ^13^C and ^15^N Resonance assignments for YdbL from *Escherichia coli* in both its apo state and in complex with the C-terminus of YnbE

**DOI:** 10.1007/s12104-026-10284-3

**Published:** 2026-11-09

**Authors:** Clare De’Ath, Georgia L. Isom, Christina Redfield

**Affiliations:** 1 Sir William Dunn School of Pathology, University of Oxford, Oxford, OX1 3RE UK; 2 Department of Biochemistry, University of Oxford, Oxford, OX1 3QU UK

**Keywords:** AsmA-like proteins, Phospholipid transport, Outer membrane biogenesis, NMR spectroscopy

## Abstract

The interaction between AsmA-like protein YdbH and YnbE constitutes one of the main putative phospholipid transport systems driving outer membrane (OM) biogenesis in *Escherichia coli*. YdbH is anchored to the IM via its N-terminus and extends into a long, periplasmic domain composed of stacked β-sheets which generates a hydrophobic groove to shield phospholipids from the aqueous periplasm. It forms a continuous cell-envelope spanning complex via its interaction with OM lipoprotein YnbE. YnbE is highly self-multimeric and forms complexes with YdbH of unknown stoichiometry. YdbL, encoded within the same operon as YdbH and YnbE, is a soluble periplasmic protein that acts in a chaperone-like role to modulate YnbE multimerisation and mediate efficient YdbH-YnbE complex assembly. NMR revealed a direct and highly conserved interaction interface between YdbL and the C-terminus of YnbE, supporting the hypothesis that YdbL can directly tune YnbE behaviour. In this report, we present the ^1^H, ^13^C and ^15^N resonance assignments for YdbL in both its apo state and in complex with a peptide derived from the C-terminus of YnbE. Analysis of the chemical shift data reveals the interaction interface for YdbL with YnbE does not result in significant changes in secondary structure but does appear to increase stabilisation of interacting α-helices. Overall, chemical shift changes observed for YdbL upon complex formation are reflective of side chain rearrangements in the peptide binding site and the interaction is confirmed with direct peptide-YdbL NOEs. Peptide-peptide short and medium range NOEs, and analysis of Hα chemical shifts, provide evidence that the bound YnbE peptide adopts an α-helical fold as predicted by *in silico* modelling of the interaction of full-length YnbE with YdbL. Together, these results strengthen directly related work underpinning YdbL function in controlling YnbE multimerisation to ensure correct complex formation with YdbH.

**Supplementary Information:**

The online version contains supplementary material available at https://doi.org/10.1007/s12104-026-10284-3.

## Biological context

The outer membrane (OM) of Gram-negative bacteria is a significant barrier, conferring intrinsic levels of resistance to antibiotics (Nikaido and Vaara 1985; Nikaido 2003). To build the OM, hydrophobic components, including phospholipids, lipopolysaccharides and proteins, must be transported from their site of synthesis in the cytoplasm, across the inner membrane (IM) to the OM whilst being continuously shielded from the aqueous periplasm (Silhavy et al. 2010). The mechanism of phospholipid transport to build and maintain the OM remained elusive until more recent work implicating the AsmA-like protein family in this role (Grimm et al. 2020; Ruiz et al. 2021; Douglass et al. 2022; Cooper et al. 2025). YdbH is one of three major AsmA-like paralogues in *Escherichia coli* considered responsible for bulk phospholipid transport (Ruiz et al. 2021; Douglass et al. 2022). YdbH is encoded in an operon with a lipoprotein, YnbE and a soluble periplasmic protein, YdbL (Kumar et al. 2024). Together, these three proteins work in concert to mediate function. YdbH, anchored to the IM, interacts with YnbE at the OM via a β-strand augmentation to form a cell envelope-spanning bridge for continuous lipophilic transport and both of these proteins are essential for function (Kumar et al. 2024). Additionally, YnbE is self-multimeric, interacting with YdbH to form complexes of unknown stoichiometry (Kumar et al. 2024). YdbL, whilst not essential for YdbH-YnbE function, modulates YnbE multimerisation in a chaperone-like role (Kumar et al. 2024; De’Ath, Kumar et al. 2026). However, it also possesses a dominant negative phenotype if present at levels relatively higher than YdbH and YnbE (Kumar et al. 2024; De’Ath, Kumar et al. 2026).

We have shown previously that a direct interaction exists between YdbL and YnbE (De’Ath, Kumar et al. 2026). In these experiments, YdbL was monitored using NMR in the presence and absence of a YnbE peptide derived from its C-terminus, the predicted binding region. Here, we present and analyse these ^1^H, ^13^C and ^15^N resonance assignments for YdbL in its apo- and YnbE peptide-bound states, comparing results to the X-ray structure of apo-YdbL and the MultiFold2-predicted model of the YdbL-YnbE complex (McGuffin et al. 2023, 2025; De’Ath, Kumar et al. 2026). Analysis of chemical shifts reveals no significant backbone conformational changes of YdbL upon YnbE binding while random coil index order parameters (RCI S^2^), which increase slightly upon binding, suggest minor stabilisation of interface helices in the complex. Additional experiments also provide more residue-specific detail of the YdbL-YnbE interaction interface. These include YdbL-peptide NOEs, and analysis of arginine and aromatic side chain chemical shifts that were previously not investigated. YdbL residues R61 and Y65, which are located in the YnbE binding site, show restricted side chain reorientation. Finally, filtered NOE data reveal peptide-specific short and medium range NOEs consistent with α-helical folding, suggesting the peptide efficiently recapitulates the expected folding profile of the YnbE C-terminus when in complex. These data consolidate findings of directly related work by De’Ath, Kumar et al. (2026) and provide more details about the structure and dynamics of residues vital for YdbL-YnbE function.

## Methods and experiments

### Protein expression and purification

YdbL without its signal peptide (residues 22–108) was expressed in T7 Express cells (NEB) via a pET21a vector. The expression vector contained an N-terminal 6xHis-tag followed by a TEV protease cleavage site. This TEV cleavage site left an N-terminal glycine (G21). For expression, overnight cultures in LB (Miller) containing 1% glucose and 100 µg/mL carbenicillin were diluted 1:15 in LB with supplemented carbenicillin before growing to an OD_600_ between 0.80 to 0.85. Cells were collected by centrifugation (4,000 x g, 15 min, room temperature), washed, and resuspended gently in 1 × M9 minimal media before collecting cells in a second centrifugation step (4,000 x g, 15 min, room temperature). After removing supernatant, cells were resuspended gently in 2 × concentrated M9 minimal media at twice the original cell density (2 L LB/1 L M9 ratio) supplemented with 1 g/L ^15^N-NH_4_Cl, and 2.5 g/L D-glucose or 2 g/L ^13^C_6_-D-glucose for ^15^N or ^13^C/^15^N labelled protein, respectively. Cells were then incubated statically at room temperature for 30 min before inducing with IPTG (final concentration 2 mM) overnight at 18 °C, 180 rpm. Cells were harvested by centrifugation and resuspended in 50 mM Tris pH 8, 300 mM NaCl, 10 mM imidazole, 10% glycerol with an EDTA-free cOmplete Protease Inhibitor Cocktail tablet (Roche). Using an Emulsiflex C3 cell disruptor (Avestin), cells were lysed in 3–4 passes at 10,000 psi and lysate spun down by centrifugation at 30,000 x g for 30 min at 4 °C. Supernatant was loaded onto Ni Sepharose 6 Fast Flow resin for 1 h at 4 °C and flow through collected via a gravity column. Protein-bound to resin was washed in 50 mM Tris pH 8, 300 mM NaCl, 30 mM imidazole and then eluted with 50 mM Tris pH 8, 300 mM NaCl, 250 mM imidazole at 4 °C. The 6xHis-tag was cleaved by addition of TEV protease (1:10 TEV:protein ratio) for 1 h at room temperature before dialysing overnight at 4 °C in 50 mM Tris pH 8, 300 mM NaCl using SnakeSkin 3.5 kDa MWCO dialysis tubing. Protein solution was then loaded again onto Ni Sepharose 6 Fast Flow resin to separate cleaved and uncleaved protein in addition to removing the His-tagged TEV protease. Purified, cleaved protein fractions were pooled and concentrated using a 3 kDa MWCO centrifugal filter (Amicon). Final samples were buffer exchanged by size exclusion chromatography (SEC) on a Superdex 75 Increase 10/300 GL (S75 10/300I) column equilibrated with 10 mM Na_2_H_2_PO_4_, 20 mM NaCl, pH 7.4 before concentrating to 180 µM for ^15^N-YdbL and 60 µM for ^13^C-^15^N-YdbL. A 1D undecoupled ^1^H NMR spectrum of the ^13^C-^15^N-YdbL sample showed that incorporation of ^13^C was only around 66% while that of ^15^N was > 95%; this was likely due to contamination of the ^13^C_6_ glucose stock. This decreased the effective concentration of the sample to 40 μM but this was still adequate to give good quality triple resonance spectra.

A peptide corresponding to residues 39 to 61 at the C-terminus of YnbE (H-EHEIIIKADKDVEELLETRSDLF-OH) was synthesised by GL Biochem to > 98% purity and provided in lyophilised form. Before experiments, the peptide was reconstituted in 10 mM Na_2_H_2_PO_4_, 20 mM NaCl buffer and the pH was adjusted to 7.4.

### NMR spectroscopy

Experiments for resonance assignment were performed using ^15^N or ^13^C/^15^N-labelled YdbL. When measuring YdbL in complex with the C-terminus of YnbE, reconstituted peptide was added to be in slight excess to the YdbL concentration. Samples were prepared with 5% D_2_O in 5 mm Shigemi tubes to a final volume of 350 µL. NMR experiments were carried out using standard triple-resonance protocols (Sattler et al. 1999; Redfield 2015) at 20 °C using 750 MHz and 950 MHz spectrometers equipped with Oxford Instruments Company magnets, Bruker Avance III HD consoles and 5 mm TCI CryoProbes.

Resonance assignments for apo-YdbL from ^15^N- and ^13^C/^15^N-labelled protein were obtained using 2D ^1^H-^15^N HSQC, ^1^H-^13^C HSQC and ^1^H-^15^N BEST-TROSY experiments and 3D ^15^N-edited NOESY-HSQC, ^15^N-edited TOCSY-HSQC, (H)CC(CO)NH and BEST-TROSY versions of HNCA, HNCACB and HNCO experiments (Schulte-Herbrüggen and Sørensen 2000; Lescop et al. 2007). Experiments for YnbE peptide-bound YdbL were conducted as per the apo-state, except that the 3D HNCACB was not collected and 3D HCCH-TOCSY and ^13^C-edited NOESY-HSQC were collected. 3D NMR data were collected with 25% non-uniform sampling (NUS) in the two indirect dimensions using standard Bruker sampling schedules throughout. 2D NMR data were processed using NMRPipe (Delaglio et al. 1995) and 3D NUS data were processed with the hmsIST software (Hyberts et al. 2012) in addition to NMRPipe.

Several peaks observed in the ^1^H-^15^N HSQC, but not in the 2D BEST-TROSY or the 3D BEST-TROSY triple resonance experiments, appeared to arise from arginine Hε-Nε and Hη-Nη. A 2D ^1^H-^15^N HSQC collected with a large ^15^N spectral width (± 35 ppm) and with the ^15^N frequency set to 80 ppm confirmed this. In order to assign these peaks to specific arginine residues, 2D ^1^H-^13^C planes of standard HNCA, and CBCANH experiments were collected to identify links to the ^13^Cγ and ^13^Cδ peaks. The ^15^N-edited TOCSY-HSQC provided links to ^1^Hγ and ^1^Hδ peaks. A 2D ^1^H-^13^C plane of an HNCO with a larger ^13^C spectral width (± 12.5 ppm centered at 171 ppm) and the ^15^N frequency set to 80 ppm was used to link the ^1^Hε to the ^13^Cζ.

Interactions between YdbL and the YnbE peptide were probed using the 3D ^15^N-edited NOESY-HSQC and the ^13^C-edited NOESY-HSQC. A 2D ^1^H-^1^H NOE with a ^15^N double half filter (Otting and Wüthrich 1989) was used to identify NOEs within the YnbE peptide in the bound state and to confirm NOEs between YdbL ^1^H^N^ and YnbE. A 2D ^1^H-^1^H TOCSY with a ^15^N filter (Ogura et al. 1996) was used to identify the ^1^H^N^, ^1^Hα and sidechain ^1^H for the YnbE peptide in the bound state. These filtered experiments were carried out with a ^15^N-labeled sample of YdbL in the presence of peptide. The incomplete ^13^C labelling of the ^13^C-^15^N sample meant that the ^15^N/^13^C filtered experiment could not be carried out for this sample. ^1^H assignments for the YnbE peptide in the free state were obtained using ^1^H-^1^H NOESY and TOCSY experiments for a sample of the peptide alone at pH 7.4.

NMR spectra were analysed and assignments recorded using CcpNmr Analysis v2.5.2 (Vranken et al. 2005). To reference ^1^H and ^13^C chemical shifts, sodium 3-(trimethylsilyl)− 1-propanesulfonate (DSS) was used whilst ^15^N chemical shifts were referenced indirectly. Specific experimental details and sample conditions can be found in the relevant BMRB deposition files.

### Secondary structure assignment and chemical shift analysis

Using the assigned chemical shifts, the ΔCα-ΔCβ secondary chemical shift differences were calculated to identify regions of secondary structure (Spera and Bax 1991; Metzler et al. 1993). Residue-specific random coil values averaged from BMRB chemical shift statistics were used (https://bmrb.io/ref_info/csstats.php?set=full&restype=aa&output=html). Additionally, TALOS-N was used in default mode using the online server to predict secondary structure (Shen and Bax 2013). Random Coil Index (RCI) order parameter values (S^2^) generated as per (Berjanskii and Wishart 2005), were taken from the TALOS-N output. ^1^Hα chemical shifts for the YnbE peptide in the free and YdbL-bound states were analysed using the Secondary Structure Propensity (SSP) method (Marsh et al. 2006). The SSP scores were calculated with an averaging window of 3 residues. Hydrogen bonds and solvent accessibility were predicted from structural models using HBPLUS v3.15 (McDonald and Thornton 1994) and NACCESS v2.11(Lee and Richards 1971; Hubbard and Thornton 1993).

### Extent of assignments and data deposition

**Fig. 1:**
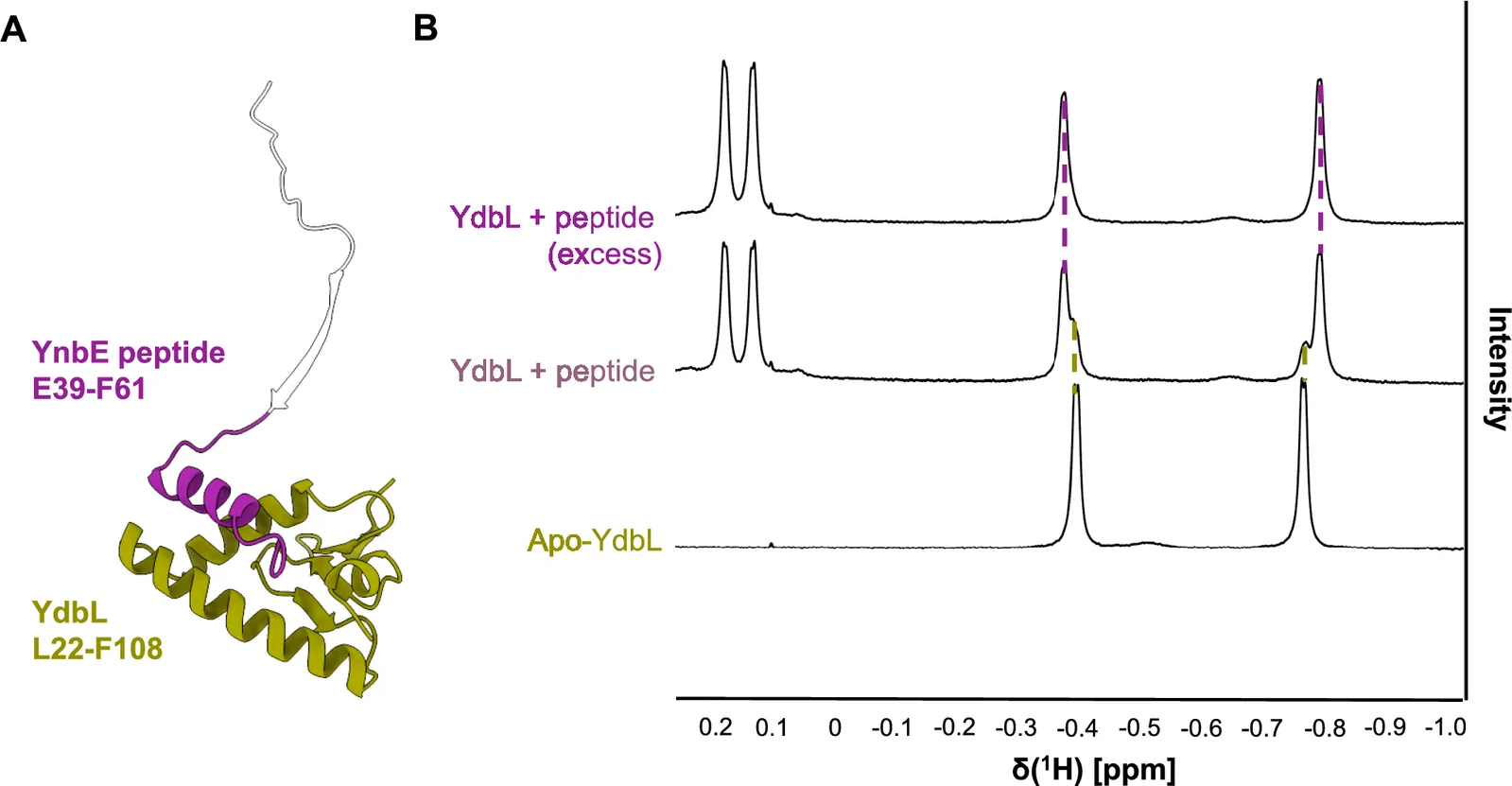
950 MHz 1D ^1^H experiments reveal YdbL binds YnbE with slow exchange dynamics. **(A)** MultiFold2 structural prediction of YdbL with YnbE showing the predicted binding site. Mature YdbL (gold, residues L22-F108) interacts with the C-terminal α-helix of YnbE (grey and plum, residues C17-F61). A custom peptide was designed (plum, residues E39-F61) to probe this interaction interface by NMR. Structural prediction information can be found in De’Ath, Kumar et al. (2026). **(B)** 950 MHz 1D ^1^H experiments showing chemical shift changes occurring at 0.2 to − 0.1 ppm. YdbL is shown with increasing additions of YnbE peptide to molar excess. Dotted lines show the chemical shifts for the methyl peaks in apo- (gold) and peptide-bound (plum) environments for YdbL + peptide

1D ^1^H experiments on unlabelled protein were used initially to probe the interaction between YdbL and the C-terminal peptide of YnbE (Fig. 1A). The upfield methyl region of the 1D spectrum shows changes upon addition of YnbE peptide. (Fig. 1B). For YdbL alone, two resolved peaks were observed at − 0.40 and − 0.76 ppm. Upon addition of less than one molar equivalent of peptide, the intensity of the two apo-state peaks decreased and two new peaks at − 0.38 and − 0.79 ppm appeared. The addition of a further aliquot of peptide resulted in the disappearance of the two apo-state peaks and an increase in intensity of the two new peaks. This confirmed the interaction of YdbL with the YnbE peptide and showed that the interaction is in slow exchange on the NMR timescale, likely indicating a high affinity interaction (low μM to nM affinity range). Interestingly, a second pair of peaks with linewidths comparable to those of YdbL appears in the spectrum at ~ 0.17 ppm; these were subsequently assigned to a pair of leucine methyl groups from the bound state of YnbE. The observation of slow exchange behaviour in the NMR spectra meant that assignments could not be directly transferred from the apo-state to the peptide-bound state via titrations and that separate triple-resonance data collection for apo- and peptide-bound YdbL was required to achieve complete assignments.

**Fig. 2:**
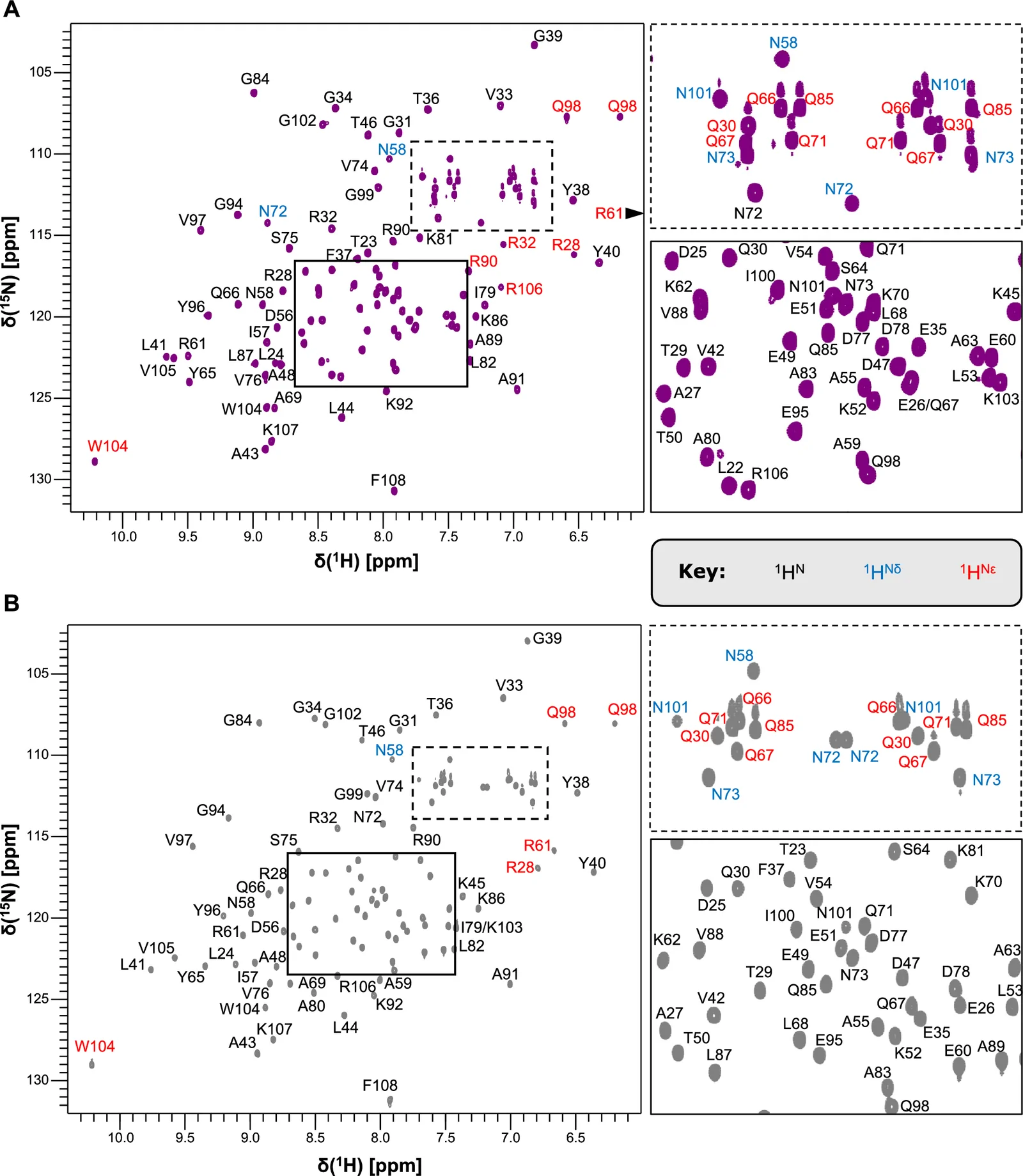
950 MHz ^1^H-^15^N HSQC spectra of YdbL in 10 mM Na_2_H_2_PO_4_, 20 mM NaCl, pH 7.4 **(A)** with YnbE peptide bound (plum) or **(B)** alone (grey). Peaks for the ^1^H^N^ (black), ^1^H^Nδ^ (blue) and ^1^H^Nε^ (red) are annotated by residue. The boxed regions of each spectrum (either unbroken or dashed) are enlarged for better labelling clarity. The^1^H^Nε^-^15^N^ε^ peak of R61 shifts upfield to 5.4 ppm in the presence of peptide (shown by arrow in panel A). The arginine peaks labeled in red correspond to the side chain ^15^N^ε^-^1^H^ε^; these are aliased in these HSQC spectra and have ^15^N chemical shifts in the region of 80–85 ppm (see also Supporting Fig. 1)

The amide ^1^H-^15^N backbone chemical shift changes previously revealed an extensive binding site for YdbL with the C-terminus of YnbE (De’Ath, Kumar et al. 2026). Peaks in the 2D ^1^H-^15^N HSQC spectra for YdbL in complex with YnbE (Fig. 2A) and alone (Fig. 2B) were well-dispersed and matched the expected number of peaks. The N-terminal glycine remaining after TEV cleavage was omitted from YdbL assignment quality statistics. Therefore, of the mature full-length (87-residue) YdbL sequence containing a single proline, we assigned all 86 expected backbone ^1^H^N^-^15^N peaks in both HSQC spectra. In addition, the side chain ^15^N-^1^H_2_ peaks of asparagine and glutamine and the tryptophan indole ^15^N-^1^H were assigned in both states.

Nearly all possible backbone ^1^Hα, ^1^Hβ, ^13^Cα, ^13^Cβ and ^13^CO assignments were obtained in both protein states as shown in Table 1. One exception was N58 ^1^Hα in the apo-state due to it probably being obscured by the water peak. We also achieved a comparable level of side chain detail for both protein states in ^1^Hγ, ^1^Hδ, ^13^Cγ and ^13^Cδ (Table 1) resonances. Aromatic side chains were assigned on the basis of ^1^H-^1^H TOCSY and ^1^H-^13^C HSQC spectra and H^N^-Hδ NOEs observed in 3D ^15^N-edited NOESY-HSQC spectra. For these side chains, 75% ^1^H and 60% ^13^C^15^N assignments were made for apo-YdbL compared to 85% ^1^H and 75% ^13^C^15^N for peptide-bound YdbL. The lower number of assignments in apo-YdbL is largely due to the apparent broadening of the aromatic resonances corresponding to Y65 (see below). Chemical shift assignments for both states of YdbL have been deposited in the BioMagResBank (https://bmrb.io/) under accession codes 53​587 (apo-YdbL) and 53​588 (YnbE peptide-bound YdbL).

**Table 1:** Extent of assignments for YdbL in apo-state and in complex with C-terminus of YnbE (peptide-YdbL)

Percent Assigned (%)						
Sample	^1^H^N^/^15^N^a^	^13^CO​​	^1^Hα/^13^Cα	^1^Hβ/^13^Cβ	^1^Hγ/^13^Cγ^b^	^1^Hδ/^13^Cδ^c^
apo-YdbL	100/100	97.70	98.94/100	93.98/100	63.04/93.55	62.50/87.80
YnbE peptide-YdbL	100/100	97.70	100/100	96.99/100	82.61/91.94	72.31/87.80

^a^ Backbone statistics for ^15^N do not include Pro nitrogens.^b^Cγ from Asp, Asn, His, Phe, Tyr, Trp do not have attached^1^H and generally are not assignedso have not been included in statistics.^c^Similarly, Cδ from Glu, Gln and Trp (δ2) do not have attached^1^H and are excluded from statistics

### Analysis of predicted secondary structure and backbone order

**Fig. 3:**
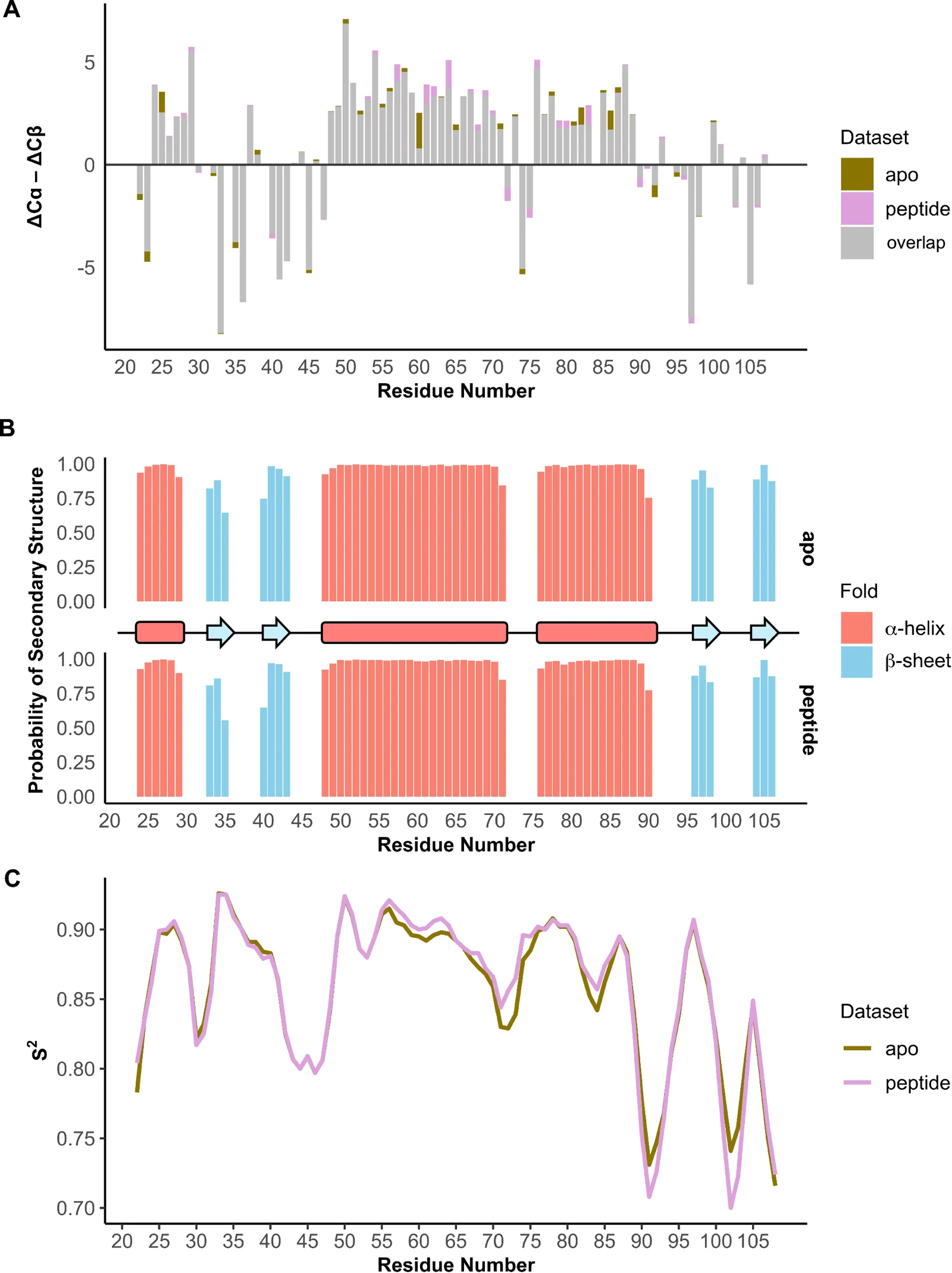
Secondary structure predictions indicate that there are no significant differences in secondary structure between apo and peptide-bound YdbL. **(A)** ΔCα—ΔCβ for both apo (gold) and peptide-bound (plum) YdbL. **(B)** TALOS-N secondary structure probability for apo (top panel) and peptide-bound (bottom panel) YdbL plotted as a function of residue number. Only secondary structure for α-helix (red) or β-sheet (blue) is shown when the probability is above 0.5. **(C)** RCI-S^2^ order parameter values of both apo (gold) and peptide-bound (plum) YdbL reveal very subtle predicted changes in protein backbone order. All residues across both states possess RCI-S^2^ values​ ​≥ 0.7, a cut-off depicting more ordered or rigid regions

The secondary structure was first analysed by calculating the ΔCα—ΔCβ secondary shift differences for both states of YdbL (Fig. 3A). Here positive and negative shift differences consistent with α-helix and β-strand secondary structure were observed, with very few differences in ΔCα—ΔCβ secondary shifts between apo and peptide-bound states, indicating that the secondary structure of the two states is very similar. There is a noticeable decrease in the ΔCα—ΔCβ difference for E60 in the peptide bound state but not for adjacent residues. E60 has a large amide backbone chemical shift change upon peptide binding (De’Ath, Kumar et al. 2026) and the reduced positive value in ΔCα—ΔCβ is likely to arise from a specific interaction predicted with the side chain of R57 from the YnbE peptide, rather than indicating a change in secondary structure.

We calculated secondary structure propensity for apo- and peptide-bound YdbL using TALOS-N (Shen and Bax 2013) (Fig. 3B). The secondary structure predictions from TALOS-N agree well with the predictions from the ΔCα—ΔCβ secondary shifts. Very little difference was observed between the TALOS-N predictions for the apo- and peptide-bound states. Notably, no change in helical probability is predicted for E60 upon peptide binding. This showed that the binding interaction between YdbL and the YnbE peptide results in only subtle backbone rearrangements and side chain interactions as opposed to significant conformational changes involving secondary structure. For apo-YdbL, TALOS-N was run with the YdbL X-ray structure resolved at 2.1 Å by De’Ath, Kumar et al. (2026) (PDB: 28OJ) for comparison. Nearly all residues showed ϕ/ψ torsion angles in ‘Good’ agreement with the X-ray model (PDB: 28OJ) with no ‘Bad’ matches. The exceptions in both states were L44 and D47, which were labelled as ‘Ambiguous’ and showed ϕ/ψ torsion angle matches in more than one region of the Ramachandran plot. Both of these residues lie at caps or boundaries to secondary structure. This may result in chemical shifts that do not match closely to those predicted for the regular torsion angles observed within α-helices or β-sheets. It is also possible that in these loop regions the structure or dynamics in solution and in the crystal may differ.

By analysing the TALOS-N derived random coil index order parameter RCI S^2^ (Fig. 3C), we also defined the predicted backbone order for YdbL. With all RCI S^2^ values above a cut-off threshold of 0.7, we confirmed that YdbL remains highly ordered regardless of an interaction with YnbE. This corroborates previous data that YdbL has a high thermal stability and an X-ray structure in which electron density is observed for all residues (De’Ath, Kumar et al. 2026). Although relatively subtle, upon YnbE-peptide binding, there is an increase in protein rigidity across residues D56-V76 of an α-helix. This helical region interacts directly with the bound peptide. This is consistent with an ~ 12 °C increase in thermal stability observed previously for the YdbL-peptide complex relative to apo-YdbL. Otherwise, the RCI S^2^ values for both states are highly similar in regions of secondary structure. Towards the C-terminus of YdbL, there are some decreases in the RCI S^2^ values for two loop regions; these residues do not interact with bound peptide. Overall, the RCI-S^2^ order parameters are in agreement with a picture of a well-ordered structure in both the apo and peptide-bound states.

### Chemical shift changes arising from the interaction of YdbL with the C-terminus of YnbE

As noted previously (De’Ath, Kumar et al. 2026), the most significant amide backbone chemical shift changes upon peptide binding were generally in good agreement with residues predicted to form the YdbL binding pocket and mediate hydrogen bonds with YnbE. However, there were a few notable exceptions, especially in the case of arginine residues. For example, although R61 and R90 both had significant amide backbone chemical shift changes in agreement with their predicted location in the binding pocket of YdbL and forming putative hydrogen bonds with YnbE, R28 did not. It was hypothesised that R28, which lies outside the two major α-helices that form the binding pocket, could still form a hydrogen bond with YnbE via its relatively long side chain and that this interaction might not perturb the backbone amide resonance. Without arginine side chain Nε-Hε assignments, assessment of this interaction was not possible.

In this study, we present more complete chemical shift assignments, especially for arginine residues. In both datasets, Hε-Nε peaks from the guanidinium side chain group for arginines were assigned in the ^1^H-^15^N HSQC. Assignments were made for R28 and R61 in the apo dataset compared to all five arginines (R28, R32, R61, R90 and R106) in the peptide-bound dataset, with chemical shift changes shown in Table 2. The observation of more Hε-Nε peaks in the peptide-bound state that were previously broadened in the apo state indicated that these could be less solvent-exposed or restricted in their side chain motion upon forming a complex with YnbE. R28 Hε-Nε peaks were observed in both states but with a notable chemical shift change upon complex formation that could result from an altered chemical environment by hydrogen-bonding to YnbE F61 (Table 2). The R61 Hε had a large upfield shift upon peptide binding which was likely a combined result of through-space ring current shifts from the aromatic ring of F61 from the peptide and additional hydrogen bonding to YnbE L60 (Table 2). The Hε-Nε peak observed for R90 in complex with YnbE was expected. This supported R90 as a highly solvent-exposed residue becoming rigidified upon complex formation via predicted hydrogen bonding to YnbE E51 located in the midsection of the α-helix (Table 2). R61 and R90 both showed chemical shift changes in their ^13^Cδ atoms in agreement with their chemical environment changing to engage the YnbE peptide. On the other hand, R32 and R106 which both have Hε-Nε peaks assigned upon peptide-binding, are not expected to become more buried but are predicted to alter their hydrogen bonding (Table 2). Therefore, this could indicate that complex formation generally rigidifies or stabilises other environments of YdbL.

**Table 2:** Chemical shift changes for arginine residues

	Hε-Nε chemical shift (ppm)		Shift change (ppm)	Relative solvent accessible area for side chain (%)		Predicted H-bonds	
	Apo	Pep		Apo	Pep	Apo	Pep
R28	6.79/ 83.92	6.54/ 83.11	0.275	20.6	11.5	R28Nε- E95Oε1 R28Nη2-E95Oε2	R28Nη1-F61OT1 R28Nη2-E35Oε1 R28Nη2-F61OT2
R32	N/A	7.08/ 82.57	N/A	19.9	30.6	R32Nη1-Q30Oε1 R32Nε-T50Oγ1 R32Nη2-D47Oδ2 R32Nη2-T50Oγ1	R32Nε-Q30Oε1 R32Nη2-Q30Oε1
R61	6.66/ 82.80	5.38/ 80.47	1.321	13.4	0.0	R61Nη1-E35Oε1 R61Nη​2-E35Oε2 R61Nη1-G39O	R61Nη2-E35Oε1 R61Nη1-E35Oε2 R61Nη​1-G39O R61Nε-L60O R61Nη2-L60O
R90	N/A	7.34/ 84.22	N/A	52.9	52.0	NONE	R90Nη1-E51Oε1
R106	N/A	7.09/ 85.19	N/A	64.9	49.5	NONE	R106Nε-G94O

The assigned Hε-Nε chemical shifts (ppm) are given for apo and peptide-bound YdbL with the combined chemical shift change (ppm) where applicable. The relative solvent accessible area (%) for each arginine total side chain was calculated using NACCESS and the side chain hydrogen bonds (H-bonds) were predicted using HBPLUS for apo-YdbL (from the X-ray model PDB: 28OJ) and peptide-bound YdbL (from the MultiFold2 structural prediction). Atoms in bold correspond to the YnbE peptide rather than YdbL. ‘OT’ refers to the terminal carboxylate group of the YnbE peptide F61

In addition, the dynamics of the Y65 aromatic ring in YdbL appears to change upon binding of the peptide. Separate ^1^H and ^13^C resonances are observed for the δ1, δ2, ε1 and ε2 atoms indicating slow flipping of the Y65 ring on the NMR timescale. More typically, aromatic rings in proteins flip rapidly, with the δ and ε atom pairs averaged into single peaks. This slow ring flipping of Y65 is consistent with this residue being located in the YnbE binding pocket of YdbL. Peptide binding is predicted to result in complete burial of Y65 with peptide residues L54 and F61 in close proximity to the Y65 aromatic ring. Interestingly, the Y65 ring hydroxyl proton is also observed in the NMR spectrum at 9.20 ppm. This group is buried and predicted to be hydrogen bonded to the backbone carbonyl of G39. In apo-YdbL, peaks for Y65 δ and ε could not be assigned due to apparent line broadening; no Y65 δ or ε peaks are identified in 2D ^1^H TOCSY, NOESY or ^1^H-^13^C HSQC spectra, or from the Y65 H^N^ in the 3D ^15^N-edited NOESY-HSQC. The absence of peaks suggests that ring flipping has become somewhat faster in the absence of YnbE peptide so that these peaks are in the intermediate exchange regime. One side of the Y65 ring has some solvent exposure in the apo-state. However, it is also possible that the δ and ε chemical shift differences have become smaller which could also shift the system into intermediate exchange; further experiments will be required to resolve this. Interestingly, the Y65 ring hydroxyl proton is observed in the apo-YdbL NMR spectrum at 9.27 ppm. The YdbL X-ray structure shows that this group is hydrogen bonded to the backbone carbonyl of G39.

The side chain of R61 is in close proximity to Y65 in both apo and peptide-bound YdbL. The perturbation of the Nε-Hε peak arising from interaction with the YnbE peptide has already been noted above. In both the apo and peptide-bound states, peaks are observed in the ^1^H-^15^N HSQC for ^1^H​η − ^15^Nη; separate ^15^Nη peaks are observed for η​1 and η2 in both states indicating restricted rotation about the Nε-Cζ​ bond. In addition, in both states four ^1^Hη​ resonances are observed indicating restricted rotation about the Cζ​-Nη​ bonds (Supporting Fig. 1). The X-ray structure of apo-YdbL shows a pair of hydrogen bonds between R61 Nη​1/2 and E35 Oε1/2 and between R61 Nη​1 and G39 O. The YnbE-YdbL model also shows these hydrogen bonds with an additional hydrogen bond between R61 Nη​2 and L60 O from YnbE. These hydrogen bonds and the interaction with Y65 are likely to be responsible for the unusual dynamics of the R61 side chain. Further experiments may lead to a more complete understanding of these side chain dynamics.

### Conformation of bound YnbE peptide and NOEs between YnbE and YdbL

In this study we have used ^15^N-labelled and ^13^C/^15^N-labelled YdbL; this has allowed nearly complete resonance assignment, analysis of chemical shifts to provide secondary structure information, and has also allowed chemical shift changes in YdbL upon peptide binding to be interpreted. On the other hand, all experiments have been carried out with unlabelled YnbE peptide which has made a detailed analysis of the peptide conformation and interactions more challenging. However, it has been possible using ^15^N-filtered NOESY and TOCSY experiments to assign a significant number of ^1^H resonances in the bound peptide, to identify short and medium range NOEs within the bound peptide and to identify NOEs between YdbL and the bound peptide. Incomplete incorporation of ^13^C into the double labelled ^13^C^15^N-YdbL sample meant that ^13^C-filtered experiments were not possible; this limited complete side chain assignment for the bound peptide and the unambiguous identification of NOEs involving aliphatic and aromatic ^1^H.

**Fig. 4:**
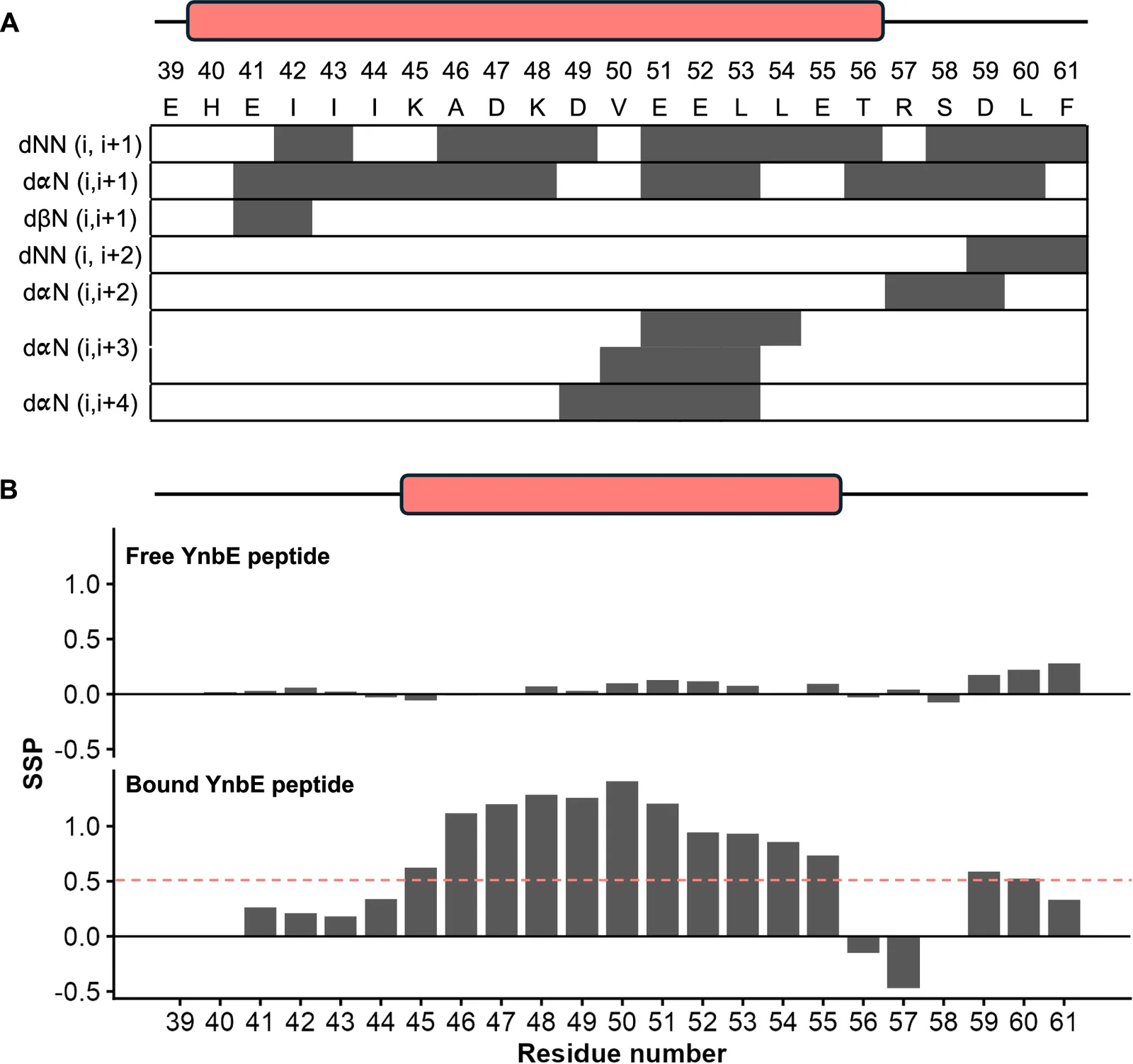
Peptide-peptide NOEs and secondary structure prediction reveals the bound state of the YnbE peptide adopts α-helical folding. **(A)** Summary of short- and medium-range backbone NOEs between peptide residues determined from assignments in the bound state with YdbL. A schematic above the residue number reveals the secondary structure prediction by MultiFold2, whereby a cylinder (salmon pink) corresponds to α-helical folding and a straight line is not predicted to have higher order secondary structure. **(B)** The secondary structure propensity (SSP) algorithm used to predict secondary structure from ^1^Hα chemical shifts. Generally, values closer to + 1 are indicative of α-helical propensity and values closer to − 1 more indicative of β-strand propensity. The dashed line (salmon pink) at 0.5 depicts significant propensity for α-helical folding. A 3-residue window was selected in the absence of other available backbone shifts in both peptide states. The schematic above the SSP plot corresponds to secondary structure predicted by the SSP algorithm

Short and medium range ^1^H-^1^H NOEs involving Hα, Hβ and H^N^ in the bound state of YnbE peptide are illustrated schematically in Fig. 4A. The sequential i to i + 1 NOEs were used to assign the backbone H^N^-Hα peaks in the TOCSY spectrum. The medium range i to i + 3 and i to i + 4 NOEs observed for residues D49 to L54 are consistent with helical secondary structure in the peptide. Because NOE analysis for the peptide was limited to ^1^H-^1^H NOEs observed in the 2D ^15^N-filtered NOESY, some potential medium range NOEs could not be assigned unambiguously due to chemical shift overlap.

Analysis of the ^1^Hα chemical shifts for the YnbE peptide in the free state in solution using the Secondary Structure Propensity (SSP) program (Marsh et al. 2006) showed no propensity for α-helical or β-strand secondary structure, indicating that the free peptide is disordered in solution (Fig. 4B). Similar analysis of the ^1^Hα chemical shifts for YnbE bound to YdbL shows significant helical propensity for residues K45 to E55 (Fig. 4B). This is consistent with the observed medium range NOEs and with the prediction of helical peptide conformation in the MultiFold2 model. However, at the N-terminus of the peptide, the α-helix is not as long as predicted in a peptide-YdbL model (De’Ath, Kumar et al. 2026). This indicates that helical folding is only stabilised for YnbE residues directly interacting with YdbL. The ^1^Hα shift analysis indicates an abrupt end to the helix at residue T56 and R57 with an increase in helical propensity at the C-terminus.

**Fig. 5:**
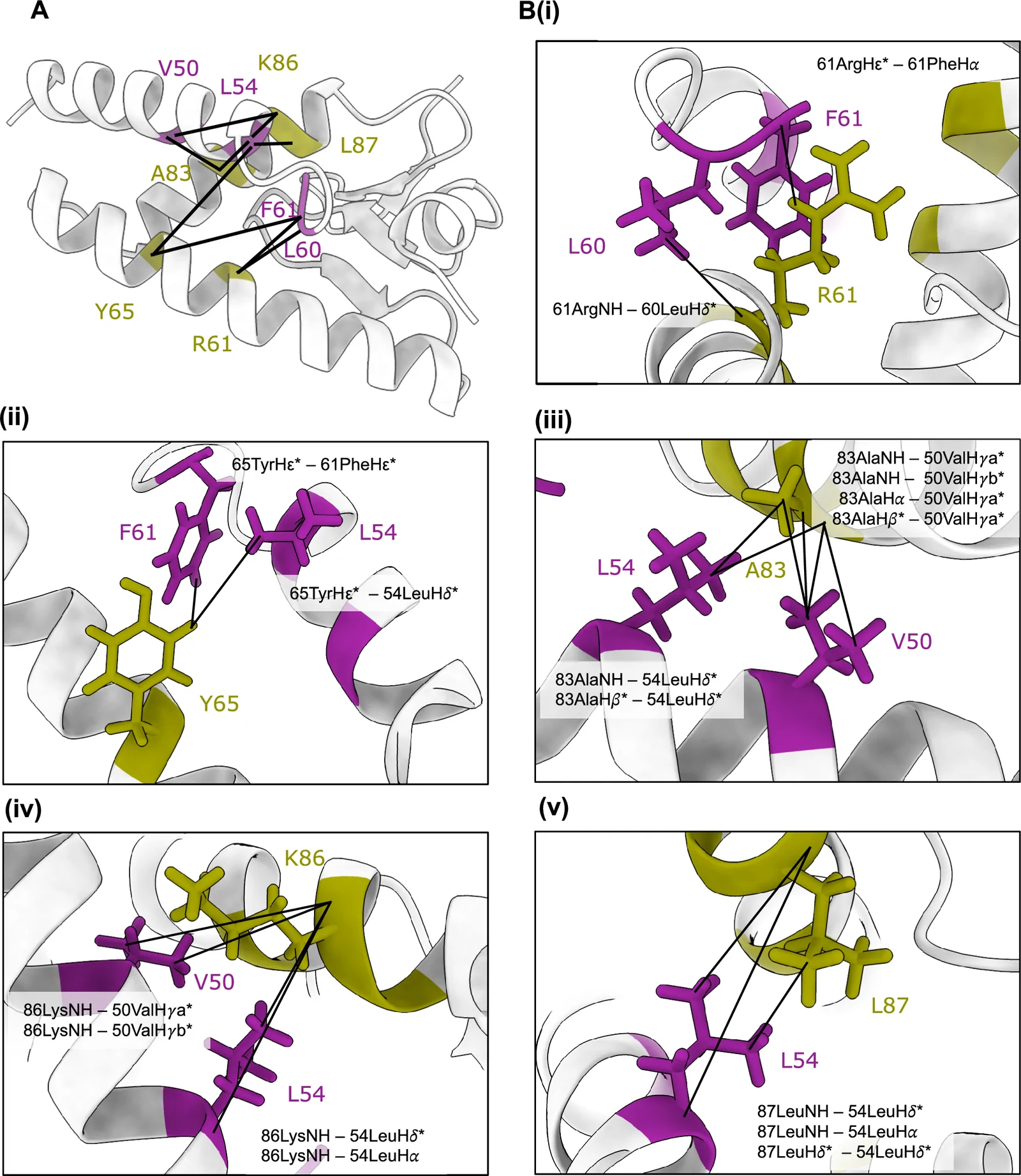
NOEs detected between YdbL and the YnbE peptide. **(A)** Summary of YdbL (gold) and YnbE peptide (plum) residues involved in NOEs (black lines) overlaid on MultiFold2 structural prediction. **(B)** Enlarged regions with NOEs detected from the YnbE peptide to residues **(i)** R61, **(ii)** Y65, **(iii)** A83, **(iv)** K86 and **(v)** L87 of YdbL. Side chains with hydrogen atoms are shown. Black lines are shown between representative atoms involved in NOEs. For methyl groups of Leucine and Valine, the lines are drawn to the carbon as the three protons rapidly average. Stereospecific assignments have not been made for the methyl groups of Leucine and Valine, and the ring protons of Tyrosine and Phenylalanine. Therefore, the lines are drawn according to the shortest distance between atom pairs in the model

The peptide-peptide NOEs and SSP predictions confirm that the YnbE peptide folds upon binding to YdbL and adopts an α-helical conformation. In addition to information about the bound peptide, the 2D filtered NOESY, 3D ^15^N-edited NOESY-HSQC and ^13^C-edited NOESY-HSQC also allowed identification of NOEs between the peptide and YdbL, providing further corroboration of residues involved in the interaction interface (Fig. 5). These NOEs were principally derived from V50, L54, L60 and F61 from the peptide, and from R61, Y65, A83, K86 and L87 of YdbL (Fig. 5). All NOEs detected between peptide and YdbL are consistent with the distances predicted within the expected binding interface from the MultiFold2 model (De’Ath, Kumar et al. 2026). These NOEs are also consistent with the residues showing some of the most significant amide backbone chemical shifts as discussed in De’Ath, Kumar et al. (2026).

## Conclusion

We report near-complete backbone chemical shift assignments of YdbL alone and in complex with a peptide derived from the C-terminus of YnbE. These assignments are integral to understanding the nature of YdbL’s direct interaction with YnbE for function as discussed previously by De’Ath, Kumar et al. (2026). Crucially, the residues mediating the YdbL-YnbE interaction interface are consistent with residues important for function in previous mutagenesis (De’Ath, Kumar et al. 2026). The high global rigidity of YdbL alone and lack of significant conformational change upon binding reflects the stable nature of YdbL on its own. The interaction interface elucidated here suggests that YdbL may indeed control YnbE self-multimerisation via steric hindrance. Future work into mechanistic details underlying YdbL’s role in controlling YnbE multimerisation and forming the YdbH-YnbE complex will be required for a holistic understanding of how this tripartite system contributes to outer membrane biogenesis.

## Supplementary Information

Below is the link to the electronic supplementary material.

Supplementary file1 (PDF 1090 KB) Fig. 1 Identification of arginine side chain Hε, Hη, Cζ, Nε and Nη for YdbL in complex with the YnbE peptide.** (A) **Part of a 950 MHz 2D ^1^H-^15^N HSQC spectrum collected with the ^15^N frequency set to 80 ppm. Five peaks corresponding to the Hε-Nε correlations for R28, R32, R61, R90 and R106 are observed in the region of 80-85 ppm in the ^15^N dimension. Two pairs of peaks in the region of 73-75 ppm in the ^15^N dimension are assigned to the Hη-Nη correlations for R61. Very distinct Hη chemical shifts are observed in each pair due to the very different environments the two Hη experience due to hydrogen bonding to the side chain of E35 and the backbone CO of G39 and L60 (from YnbE). The assignment of these peaks to R61 is confirmed by the observation of NOEs between R61 Hε and the Hη. The observation of two distinct Nη and four distinct Hη peaks for R61 is consistent with restricted rotation about the Nε-Cζ and Cζ-Nη bonds, respectively. **(B) **Part of a 950 MHz 2D ^1^H-^13^C plane of an HNCO spectrum collected with the ^15^N frequency set to 80 ppm. The four peaks observed correspond to the Hε-(Nε)-Cζ correlations for R28, R32, R90 and R106. The Hε-(Nε)- Cζ peak for R61 is not observed in this spectrum due to poor suppression of the water peak and reduced signal-to-noise due to the lower protein concentration in the double-labelled sample

## Data Availability

All resonance assignments for YdbL in apo- or YnbE-bound states are deposited in the BMRB under accession codes 53​587 and 53​588 respectively. ^1^​H resonance assignments for bound YnbE peptide are also included in the 53588 entry.
