# Supplementary material for: 1H, 13C and 15N Resonance assignments for YdbL from Escherichia coli in both its apo state and in complex with the C-terminus of YnbE
Source: Biomol NMR Assign. 2026 Nov 9;20(1):36. doi: 10.1007/s12104-026-10284-3 (PMC13748024; doi:10.1007/s12104-026-10284-3)
Supplement: Supplementary file 1 [file 12104_2026_10284_MOESM1_ESM.pdf]

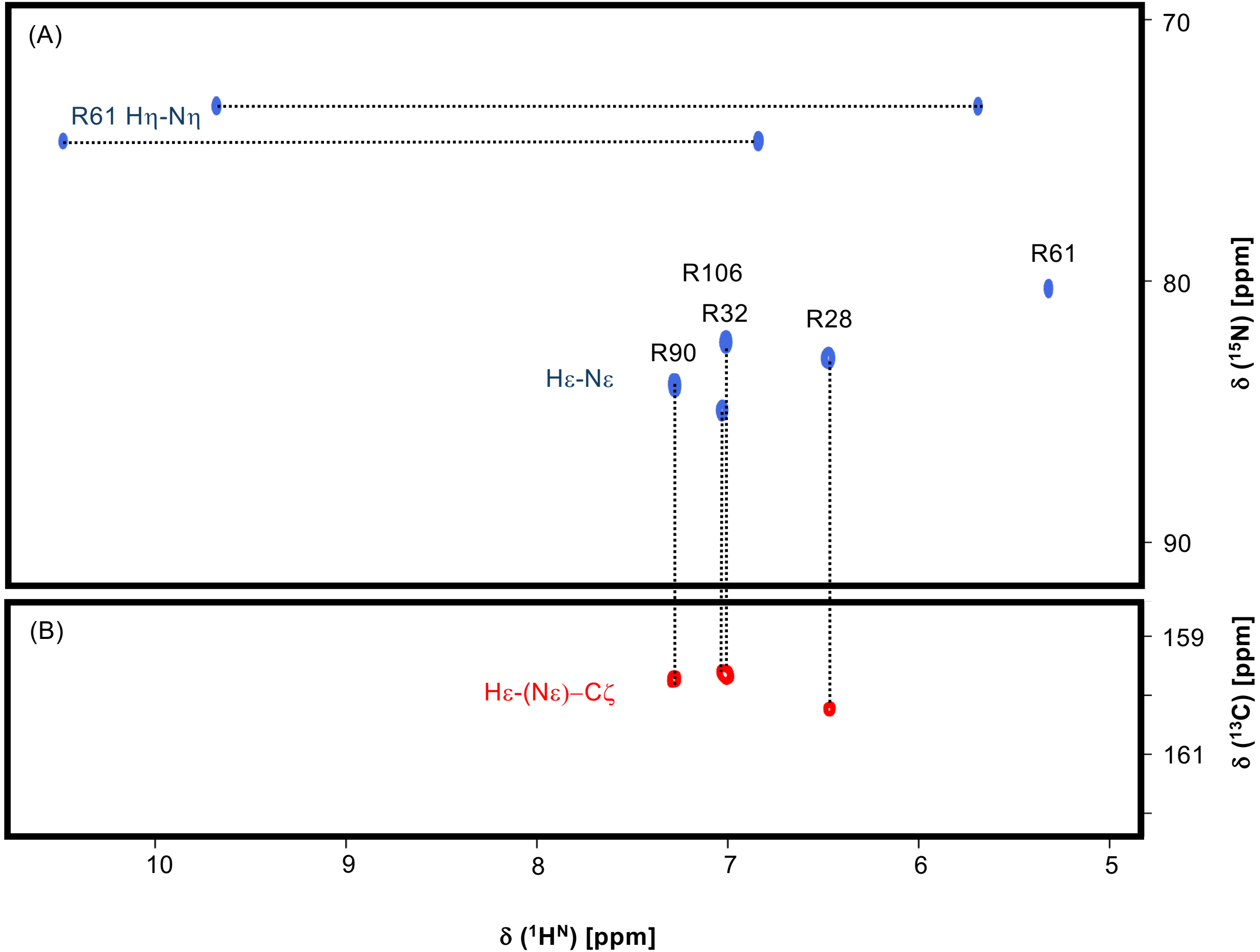
(A)
R61 Hη-Nη
R61
R106
R32
R28
R90
Hε-Nε
(B)
Hε-(Nε)–Cζ
70
80
90
159
161
10
9
8
7
6
5
δ (¹⁵N) [ppm]
δ (¹³C) [ppm]
δ (¹Hᴺ) [ppm]
